# Präventive Verhaltensweisen zum Schutz vor einer Infektion mit SARS-CoV-2 bei Menschen mit gesundheitlicher Vulnerabilität

**DOI:** 10.1007/s11553-022-00989-3

**Published:** 2022-11-08

**Authors:** Lara Schaedel, Kevin Dadaczynski

**Affiliations:** 1 Fachbereich Gesundheitswissenschaften, Hochschule Fulda, Leipziger Straße 123, Fulda, Deutschland; 2 Zentrum für Angewandte Gesundheitswissenschaften, Leuphana Universität Lüneburg, Lüneburg, Deutschland

**Keywords:** COVID-19, Gesundheitskompetenz, Pandemie, Gesundheitliche Risikofaktoren, Protektives Verhalten, COVID-19, Health literacy, Pandemic, Health risks, Protective behavior

## Abstract

**Hintergrund:**

Trotz gesundheitlicher Risiken, die für alle Menschen von SARS-CoV‑2 („severe acute respiratory syndrome coronavirus 2“) ausgehen, weisen Bevölkerungsgruppen mit gesundheitlicher Vulnerabilität ein erhöhtes Gefährdungsprofil auf. Zu den Risikogruppen für schwere COVID-19-Verläufe („coronavirus disease 2019“) gehören Personen ab 50 Jahren, Raucher*innen, adipöse Personen sowie Menschen mit bestimmten Vorerkrankungen. Gerade für diese Bevölkerungsgruppe ergibt sich ein erhöhter Schutzbedarf. Inwiefern sich das Schutzverhalten von Personen mit hoher im Vergleich zu Personen mit geringer gesundheitlicher Vulnerabilität unterscheidet, ist bislang wenig untersucht.

**Methode:**

Es wurde eine onlinebezogene Querschnittstudie mit einem „convenience sample“ von *n* = 210 in Deutschland lebenden Personen im Alter ab 18 Jahren realisiert. Es wurden subjektive Selbsteinschätzungen zur Anwendung von Infektionsschutzmaßnahmen sowie der eigenen Informationssuche und -zufriedenheit bezüglich der Pandemie und der COVID-19-bezogenen Gesundheitskompetenz (GK) erfasst. Die Analyse erfolgte uni-, bi- sowie multivariat, wobei für alle Analysen ein Signifikanzlevel von *p* < 0,05 festgelegt wurde.

**Ergebnisse:**

Über alle erhobenen Schutzverhaltensweisen hinweg weisen die Befragten eine insgesamt hohe Compliance (84 %) auf. Während das Tragen einer Maske (96 %) und das Vermeiden von privaten Reisen und Händeschütteln (95 %) die höchste Zustimmung erhielten, berührten 47 % der Befragten ihr Gesicht häufig mit ungewaschenen Händen. Jüngere (35 % bis 29 Jahre) sowie Personen mit eingeschränkter Gesundheitskompetenz (28 %) zeigen sowohl in den bivariaten und multivariaten Analyse signifikant häufiger ein weniger ausgeprägtes Schutzverhalten. Hingegen ließen sich differenziert nach Anzahl gesundheitlicher Risikofaktoren keine Unterschiede im präventiven Schutzverhalten feststellen

**Schlussfolgerung:**

Es konnte ein hohes Niveau der Anwendung von präventiven Schutzmaßnahmen bei Befragten dieser Studie ermittelt werden. Sowohl in den uni- als auch bi- und multivariaten Analysen ist die gesundheitliche Vulnerabilität nicht mit einem stärker ausgeprägten Schutzverhalten assoziiert. Insbesondere für Personen mit eingeschränkter coronaspezifischer Gesundheitskompetenz sind Defizite im Infektionsschutz erkennbar, weshalb die zielgruppenspezifische Aufklärung weiter priorisiert werden muss.

## Abstract (en)

**Background:**

Severe acute respiratory syndrome coronavirus type 2 (SARS-CoV-2) entails health-related risks for all humans, especially for vulnerable people. People at the age of 50 or older, smokers and people with obesity and comorbidities are at higher risk for a severe coronavirus disease 2019 (COVID-19) course. For this population group in particular, there is an increased need for protection. The extent to which protective behavior differs between individuals with high and low health vulnerability has been little studied so far.

**Methods:**

An online cross-sectional study was conducted including a convenience sample of 210 individuals aged 18 years and older living in Germany. In addition to infection protection measures, information seeking behavior and COVID-19-related health literacy were assessed. Univariate, bivariate, and multivariate analyses were performed, with a significance level of *p* < 0.05 for all analyses.

**Results:**

Across all ages and levels of education, respondents showed a high level of compliance with infection protection measures (84%). While wearing a mask (96%) and avoiding travelling and shaking hands (95%) were used most frequently, touching one’s face with unwashed hands were reported by 47%. People aged under 30 years (35%) as well as those with limited health literacy (28%) showed significantly lower levels of protective behaviors in bivariate and multivariate analyses. However, stratified by the number of health vulnerabilities no differences in preventive protective behavior could be found.

**Conclusion:**

A high level of use of preventive protective measures was identified among respondents in this study. In univariate, bivariate and multivariate analyses, health-related vulnerabilities are not associated with higher levels of protective behavior. Especially people with limited COVID-19-related health literacy show deficits in protective behaviors. There is a special need of target-group-specific information.

## Hintergrund und Fragestellung

Das erstmals im Dezember 2019 entdeckte Coronavirus (SARS-CoV‑2, „severe acute respiratory syndrome coronavirus 2“) erfuhr eine rasche, weltweite Verbreitung. In Deutschland ist im zeitlichen Verlauf eine hohe jahreszeitliche Variabilität der Fallzahlen erkennbar, die als Infektions- bzw. Erkrankungswellen bezeichnet werden. Nach Bekanntwerden erster Fälle im Frühjahr 2020 und zunächst steigenden Infektionszahlen wurden in den Sommermonaten 2020 und 2021 jeweils geringere Fallzahlen registriert, während mit Beginn des Herbstes 2020 und 2021 ein exponentieller Anstieg zu verzeichnen war. Dabei sind Hospitalisierungen und Todesfälle in den höheren Altersstufen gegenüber der Allgemeinbevölkerung deutlich häufiger [13].

Deutschlandweit verstarben insgesamt 1,54 von 100.000 mit SARS-CoV-2-Infizierten [14]. Innerhalb der Bevölkerung ergeben sich unterschiedliche Gefährdungsprofile. Ältere Personen ab 50 Jahren, Raucher*innen, adipöse Menschen sowie Personen mit Vorerkrankungen des Herz-Kreislauf-Systems, der Leber oder Niere, Krebserkrankungen, Diabetes mellitus und krankheits- oder medikamentös bedingter Immunschwäche werden durch das Robert Koch-Institut (RKI) als besonders vulnerabel bezüglich einer Infektion mit SARS-CoV‑2 eingestuft [12].

Zum Schutz vor einer Infektion mit SARS-CoV‑2 werden verschiedene Maßnahmen unter der Bezeichnung „AHA + L“-Regel [1, 13] empfohlen. Dazu zählen:

- Mindestabstand von 1,5 m zu anderen Personen einhalten,
- regelmäßig Hände waschen (mindestens 20 s) bzw. desinfizieren,
- Gesicht nicht mit ungewaschenen Händen berühren,
- Händeschütteln vermeiden,
- Maske tragen,
- auf private Treffen verzichten/Reisen vermeiden,
- Orte mit vielen Menschen meiden,
- Räume regelmäßig lüften.

Erste internationale Studien untersuchten einen möglichen Zusammenhang von Vulnerabilität und wahrgenommenem Infektionsrisiko mit dem Schutzverhalten. Yildirim et al. (2020) befragten hierzu im Frühjahr 2020 ein „convenience sample“ von 4536 Türk*innen. Als am häufigsten genutzte Maßnahme wird u. a. das Händewaschen genannt. Frauen haben eine signifikant höher wahrgenommene Vulnerabilität (erfasst über eine 5‑stufige Skala zur Selbsteinschätzung), ein höheres subjektives Erkrankungsrisiko und mehr coronabezogene Angst als Männer. Nach Kontrolle demografischer Faktoren erwiesen sich die gesundheitliche Vulnerabilität, das wahrgenommene Ansteckungsrisiko und die coronabezogene Angst als relevante Prädiktoren des Präventionsverhaltens [21].

Das Überangebot und die rasche Verbreitung von evidenzbasierter sowie Mis- und Desinformation über etablierte und soziale Medien begleitend zur Pandemie wird als Infodemie bezeichnet [6]. Die Ergebnisse einer im März 2020 durchgeführten Studie mit einer Gelegenheitsstichprobe zeigen, dass > 75 % der Befragten im Alter von 14 bis 84 Jahre eine Stunde und mehr Zeit aufwenden, um Informationen über SARS-CoV‑2 zu recherchieren [18]. Dabei gaben 39 % an, sich durch die Medienberichterstattung verunsichert zu fühlen.

Sowohl die Menge als auch die qualitative Heterogenität gesundheitsbezogener Informationen erfordert eine ausreichende Fähigkeit der Erschließung und des angemessenen Umgangs mit Informationen [9, 20]. Unter der Bezeichnung Gesundheitskompetenz wird das Wissen, die Motivation und Fähigkeit subsummiert, gesundheitsbezogene Informationen finden, verstehen, kritisch bewerten und für die Widerherstellung, Erhaltung und Förderung der Gesundheit einsetzen zu können.

In ihrer repräsentativen Studie von in Deutschland lebenden Menschen ab 16 Jahren stellen Okan et al. für etwa 50 % der Befragten eine eigeschränkte coronaspezifische Gesundheitskompetenz fest. Auch wenn ein Großteil der Befragten angab, sich gut über die Pandemie informiert zu fühlen, berichteten etwa 48 % Schwierigkeiten, die Vertrauenswürdigkeit medialer Informationen einzuschätzen. Dabei waren besonders Personen mit einer niedrigen COVID-19 („coronavirus disease 2019“)-bezogenen Gesundheitskompetenz häufiger von informationsbezogenen Verunsicherungen betroffen [9].

Auch berichteten 30 % der in einer bundesweiten Onlinestudie befragten Studierenden in einer frühen Phase der Coronapandemie von Schwierigkeiten im Finden gesundheitsbezogener Informationen im Internet [3]. Dabei stellte die Einschätzung der Vertrauenswürdigkeit für 42,3 % der Studierenden die größte Herausforderung dar [3].

Im Hinblick auf das Schutzverhalten zeigen deutsche Studien eine sehr hohe Bereitschaft zur Einhaltung von Maßnahmen zum Schutz vor SARS-CoV‑2 in der Bevölkerung, wie vermehrte Handhygiene, das Vermeiden privater Treffen und Reisen und das Einhalten eines Abstands zu anderen Personen. Für die meisten präventiven Schutzverhalten geben ≥ 80 % der Befragten an, diese anzuwenden. Dabei wurde jedoch nicht zwischen Personen mit hoher und geringer Vulnerabilität unterschieden [2, 3, 18]. Differenziert nach soziodemografischen Faktoren und der Gesundheitskompetenz zeigen sich in bisherigen Studien Unterschiede in der Ausprägung des Schutzverhaltens. Ein niedriger Bildungsstand gilt als ein Risikofaktor für das Anpassen der Erwerbssituation an die pandemische Lage: so waren Personen mit geringem Bildungsstand seltener in der Lage z. B. im Homeoffice zu arbeiten. Zudem vermieden Personen mit niedrigem formalem Bildungsstand private Treffen seltener als jene mit hohem Bildungsstand. Frauen hielten sich mit Ausnahme des Tragens einer Maske besser an die empfohlenen Schutzmaßnahmen [3]. Zudem zeigen sich Unterschiede hinsichtlich des Alters, wobei ältere Menschen in höherem Ausmaß protektives Verhalten anwendeten [1]. In Hinblick auf die Gesundheitskompetenz ließen sich in einer US-amerikanischen Studie Assoziationen mit der Einhaltung von verhaltensbasierten Infektionsschutzmaßnahmen bei Studierenden nachweisen. Studierende mit einer höheren allgemeinen und digitalen Gesundheitskompetenz wiesen eine höhere Compliance mit den erfassten Schutzverhaltensweisen (Tragen einer Maske, Hände waschen, „social distancing“, „staying home“) auf [11].

Verschiedene nationale Studien erforschen protektives Verhalten während der Pandemie auch im Zusammenhang mit dem wahrgenommenen Infektionsrisiko oder der Gesundheitskompetenz. Betsch et al. erfassen seit März 2020 u. a. die kognitive und affektive Risikowahrnehmung und protektives Schutzverhalten mittels Panelbefragung im zeitlichen Verlauf [2]. Lüdeke und Knesebeck erfassten in ihrer Querschnittsbefragung die Anwendung protektiver Verhaltensweisen [8]. Beide differenzieren nach soziodemografischen Merkmalen. Schulze et al. erfragten verschiedene Ansichten der Bevölkerung in der Pandemie, darunter die Einhaltung von Infektionsschutzmaßnahmen [18]. In diesen 3 Studien bleibt jedoch eine nach Vulnerabilität differenzierte Analyse protektiven Verhaltens aus. Es fehlt ein direkter Vergleich des Verhaltens von Personen mit und ohne Vulnerabilität für einen schweren Verlauf der COVID-19-Erkrankung. Vor diesem Hintergrund soll die vorliegende Studie einen Beitrag dazu leisten, Unterschiede im protektiven Verhalten von Personen mit und ohne gesundheitliche Vulnerabilität zu erkennen sowie den Zusammenhang mit soziodemografischen Faktoren und der Gesundheitskompetenz aufzuzeigen.

Es ergeben sich folgende Forschungsfragen:

1. Wie ausgeprägt ist das protektive Verhalten zum Schutz vor SARS-CoV‑2 bei Erwachsenen mit hoher und geringer gesundheitlicher Vulnerabilität?
2. Wie stehen soziodemografische Faktoren, gesundheitliche Vulnerabilitäten und die COVID-19-bezogene Gesundheitskompetenz mit dem Schutzverhalten vor SARS-CoV‑2 in Zusammenhang?

## Studiendesign und Untersuchungsmethoden

### Feldzugang und Datenbasis

Zur Beantwortung der Forschungsfragen wurde vom 20.11. bis 20.12.2020 eine quantitative Querschnittserhebung mit einer Gelegenheitsstichprobe („convenience sample“) von in Deutschland lebenden Personen ab 18 Jahren durchgeführt. Die Rekrutierung von Befragungsteilnehmenden erfolgte zum einen über soziale Medien. Zum anderen wurden alle Gruppen der Selbsthilfe Hessen per E‑Mail um Einladung ihrer Mitglieder gebeten und Arztpraxen persönlich als Anlaufstellen für gesundheitlich vulnerable Personengruppen um Mithilfe ersucht. Hierfür wurde ein Flyer erstellt, der über die Ziele, Inhalte sowie Herkunft der Studie informierte sowie einen QR-Code und einen Link mit Direktzugriff auf die Befragung enthielt. In den Einladungen per E‑Mail, die über die Selbsthilfegruppen versendet wurden, konnte die Befragung über den Link direkt aufgerufen werden. Vor dem Ausfüllen des Fragebogens mussten die Befragten ihr Einverständnis zur Teilnahme und zur Verarbeitung ihrer Daten für wissenschaftliche Zwecke erklären. Um den Rücklauf zu erhöhen, wurde nach Empfehlungen von Fan et al. [4] und Saleh et al. [15] ein prägnanter Fragebogen erstellt und vor Beginn der Befragung ein Einladungsschreiben per E‑Mail sowie Erinnerungen an die Befragungsteilnahme bzw. -beendigung zwei und eine Woche vor Ende des Erhebungszeitraums verendet. Die Durchführung der Befragung wurde mittels Pretest in mehreren Internetbrowsern getestet und die Daten testweise in SPSS importiert und ausgewertet, um Datenverlust aus technischen Gründen zu minimieren. Es wurde auf die strikte Einhaltung von Datensicherheit und Verschwiegenheit geachtet und zu Beginn der Befragung darauf verwiesen.

### Variablenbeschreibung

Der für die Erhebung konzipierte Fragebogen umfasste 25 Fragen, deren Beantwortung durchschnittlich etwa 10 Min. in Anspruch nahm. Als *soziodemografische Variablen* wurden das Geschlecht (weiblich, männlich, divers), das Alter (18–29, 30–39, 40–49, 50–59 und 60 Jahre und älter) und der Schulabschluss (ohne Abschluss, Förder‑, Haupt‑, Realschule, Fachhochschulreife, Abitur) erfasst. Für die bivariate Auswertung wurde eine Kategorisierung des Alters in zwei Gruppen (≤ 29 Jahre versus ≥ 30 Jahre) vorgenommen. Zudem wurde die Zugehörigkeit in Anlehnung an die durch das RKI benannten *Risikogruppen für schwere COVID-19-Verläufe* [12] erfasst. Hierfür wurden die fünf zum Erhebungszeitpunkt bekannten Risiken (Alter ab 50 Jahren, vorliegende Grunderkrankung, Adipositas, Raucherstatus und Immunsuppression) erhoben. Als relevante Grunderkrankungen wurden in Anlehnung an die Formulierung des RKI Herz-Kreislauf‑, Leber‑, Nieren‑, Krebs- und pulmonale Erkrankungen gewählt. Die Befragten wurden um eine Angabe zum Zigarettenkonsum (Raucher vs. Nichtraucher) gebeten. Eine spezifische Erfassung der Konsumhäufigkeit erfolgte nicht. Für die Analysen wurden die von den Befragten genannten Risiken summiert und drei Kategorien gebildet (0 gesundheitliche Risiken, 1 gesundheitliches Risiko, ≥ 2 gesundheitliche Risiken).

Die Erfassung der *Häufigkeit des umgesetzten protektiven Verhaltens* erfolgte in Anlehnung an die COSMO-Studie. Es wurden 10 der 20 Items aus der 19. COSMO-Welle ausgewählt (u. a. mindestens 20 s Hände waschen, Hände desinfizieren, Abstand von mindestens 1,5 m einhalten, Mund-Nasen-Schutz tragen, private Reisen und Treffen vermeiden), die mit Hilfe einer sechsstufigen Antwortskala „immer“ bis „nie“ bzw. „trifft nicht zu“ beantwortet werden konnten. Für die weitere Datenanalyse wurden die Antworten dichotomisiert (0 = nie/selten/manchmal, 1 = häufig/immer) und ein Summenindex gebildet (0–10). Anhand der Verteilung des Summenwertes wurden zwei Gruppen gebildet (≤ 7 vs. ≥ 8 protektive Verhaltensweisen).

Die Operationalisierung der *COVID-19-bezogenen GK* erfolgte auf Grundlage des von Okan et al. (2020) adaptierten und erweiterten HLS-EU-Q16-Instruments [9]. Die Skala umfasst insgesamt 22 Items, die von den Befragten auf einer vierstufigen Antwortskala („sehr einfach“ bis „sehr schwierig“) beurteilt werden konnten. Ein Beispielitem lautet „Ein(zu)schätzen, welche Schutzmaßnahmen ich gegen eine Ansteckung mit dem Coronavirus ergreifen sollte“ ist für mich „sehr einfach/einfach/schwierig/sehr schwierig“. Die Reliabilität ist für diese Skala als sehr gut zu bewerten (α = 0,94). Für die Kategorisierung nach Okan et al. wurde anhand des über alle Items erreichneten arithmetischen Mittels die Einteilung in ≤ 2,5 = inadäquate, > 2,5 bis < 3 = problematische und ≥ 3 = ausreichende GK vorgenommen. Die Kategorisierung wurde nachfolgend weiter zusammengefasst: „ausreichend“ und „limitiert“ (Summe aus „inadäquat“ und „problematisch“).

### Stichprobenbeschreibung

Die Verteilung der soziodemografischen Merkmale ist in Tab. 1 zu finden. Insgesamt konnten Daten von *n* = 210 Befragten gewonnen werden. Der überwiegende Teil der Befragten ist weiblich (66,2 %) und unter 40 Jahre alt (50,5 %). Einen Hauptschulabschluss als höchsten Schulbildungsgrad geben 7,6 % der Befragten an, während 28,1 % einen Realschulabschluss und 64,3 % die Allgemeine oder Fachhochschulreife nennen.

**Tab. 1:** Stichprobenbeschreibung (gültige Prozente, fehlende Werte unberücksichtigt)

	Häufigkeiten	
	Anteilig (%)	Absolut (*n*)
*Geschlecht*		
Männlich	33,8	71
Weiblich	66,2	139
*Alter (Jahre)*		
18–29	32,4	68
30–39	18,1	38
40–49	8,6	18
50–59	16,2	34
60 und älter	24,8	52
*Schulbildung*		
Hauptschule	7,6	16
Realschule	28,1	59
Abitur/Fachhochschulreife	64,3	135
*Gesamt*	*100*	*210*

### Statistische Analysen

Zur statistischen Auswertung wurden Häufigkeitstabellen (univariate Analyse) sowie Kreuztabellen und χ^2^-Tests nach Pearson (bivariate Analyse) herangezogen. Weiterhin wurden binär-logistische Regressionsmodelle berechnet (multivariate Analyse). Die Interpretation wurde anhand der Chancenverhältnisse (Odds Ratio [OR]) mit 95 %-Konfidenzintervall (‑KI) vorgenommen. Hierzu wurden die unabhängigen Variablen Geschlecht, Alter, Schulbildung, Anzahl Risikofaktoren und COVID-19-bezogene Gesundheitskompetenz einzeln und in einem Gesamtmodell hinsichtlich einer geringeren Anwendung von protektiven Verhaltensweisen betrachtet. Die in Tab. 3 zuerst aufgeführte Gruppe ist die Referenzkategorie (OR = 1,00), zu der die übrigen im Kontrast stehen. In den beiden linken Spalten befinden sich die Ergebnisse der Einzelmodelle (M1a–M1f) als OR und für das 95 %-KI, die zuerst beschrieben werden. In der rechten Spalte sind die Werte des Gesamtmodells (M2) zu finden.

Sämtliche statistische Analysen wurden mit der Software IBM® SPSS® Statistics Version 25 (IBM Corp., Armonk, NY, USA) durchgeführt. Für alle Analysen wurden ein Signifikanzniveau von *p* < 0,05 festgelegt.

## Ergebnisse

In der Erfassung der selbstberichteten Zugehörigkeit zu Risikogruppen für schwere Krankheitsverläufe sind das Alter über 50 Jahre (33 %) und eine bestehende Grunderkrankung (32 %) die am häufigsten angegebenen Risiken. Es folgen vorhandene Immunsuppression (17 %), Raucherstatus (16 %) und Adipositas (9 %). Jeweils etwa ein Drittel der Befragten berichten kein gesundheitliches Risiko (37,6 %) oder ein gesundheitliches Risiko (34,8 %), während etwa 28 % zwei oder mehr gesundheitliche Risiken für einen schweren COVID-19-Verläufe angeben.

**Abb. 1:**
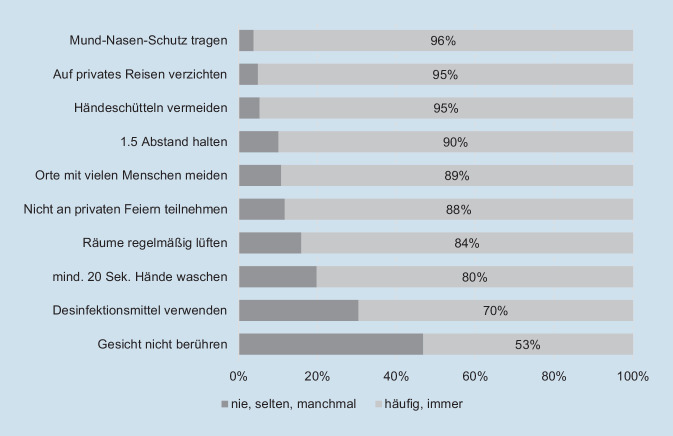
Häufigkeit des individuellen Verhaltens zum Schutz einer Ansteckung mit dem Coronavirus (*n* = 203–209)

In Abb. 1 sind die prozentualen Häufigkeiten zur Einhaltung verschiedener Schutzverhaltensweisen aufgeführt. Insgesamt zeigt sich ein sehr hohes Maß der Anwendung empfohlener Schutzverhaltensweisen. Einen Mund-Nasen-Schutz tragen 96 % der Befragten häufig oder immer. Zudem werden private Reisen und Händeschütteln von 95 % laut Selbstangaben der Befragten vermieden. Die geringste Zustimmung zeigt sich hingegen für das Berühren des Gesichts mit ungewaschenen Händen (53 %).

„Sehr gut“ oder „gut“ fühlen sich 80 % der Befragten über die Pandemie informiert. Verunsichert äußern sich 57 % der Teilnehmenden, während 42 % angeben, nicht durch die Informationslage verunsichert zu sein.

Signifikante Unterschiede in der Anzahl der häufig und immer umgesetzten Schutzverhaltensweisen finden sich für das Alter sowie die Gesundheitskompetenz, nicht jedoch für das Geschlecht, die Schulbildung und die Anzahl an gesundheitlichen Risikofaktoren (Tab. 2). Dabei berichten jüngere Befragte (≤ 29 Jahre) im Vergleich zu älteren Befragten deutlich seltener das häufige Umsetzen von acht und mehr Schutzverhaltensweisen (χ^2^ [df = 2] = 10,022, *p* < 0,01). Differenziert nach COVID-19-bezogener Gesundheitskompetenz zeigt sich, dass Befragte mit einer ausreichenden GK häufiger ≥ 8 Präventionsmaßnahmen ergreifen als jene mit eingeschränkter GK (χ^2^ [df = 1] = 3,748,* p* < 0,05).

**Tab. 2:** Individuelles Schutzverhalten stratifiziert nach Soziodemografie, gesundheitlichem Risiko und Gesundheitskompetenz (*n* = 197–203)

	Präventives Schutzverhalten	
	≥ 8 Schutzverhalten *n* (%)	≤ 7 Schutzverhalten *n* (%)
*Geschlecht*	n. s.	
Männlich	49 (71,0)	20 (29,0)
Weiblich	109 (81,3)	25 (18,7)
*Alter*	χ^2^ (df = 2) = 10,022,* p* < 0,01	
≤ 29 Jahre	42 (64,6)	23 (35,4)
30 bis 49 Jahre	44 (81,5)	10 (18,5)
≥ 50 Jahre	72 (85,7)	12 (14,3)
*Schulbildung*	n. s.	
Hauptschule	12 (85,7)	2 (14,3)
Realschule	46 (83,6)	9 (16,4)
Abitur/Fachhochschulreife	100 (74,6)	34 (25,4)
*Risikogruppe*	n. s.	
0 Risiken	56 (73,7)	20 (26,3)
1 Risiko	54 (75,0)	18 (25,0)
≥ 2 Risiken	48 (87,3)	7 (12,7)
*COVID-19-bezogene Gesundheitskompetenz*	χ^2^ (df = 1) = 3,748,* p* < 0,05	
Ausreichend	79 (83,2)	16 (16,8)
Eingeschränkt	73 (71,6)	29 (28,4)
*Gesamt*	*158 (77,8)*	*45 (22,2)*

*COVID-19* „coronavirus disease 2019“

Die Tab. 3 zeigt die Ergebnisse für die binär-logistischen Regressionsmodelle für eingeschränktes Schutzverhalten (d. h. ≤ 7 häufig oder immer ausgeübte Verhaltensweisen). Diese wurden zunächst separat für jede unabhängige Variable und anschließend im Gesamtmodell unter Berücksichtigung aller Variablen berechnet. Im Hinblick auf die Einzelmodelle ergeben sich für das Geschlecht (M1a), die Schulbildung (M3a) und die Anzahl der gesundheitlichen Risikofaktoren (M4a) keine signifikanten Assoziationen mit einer verringerten Anzahl an umgesetzten COVID-19-bezogenen Schutzverhaltensweisen. Die Befunde zum Alter deuten darauf hin, dass Personen bis einschließlich 29 Jahren ein mehr als 3fach erhöhtes Risiko aufweisen, ≤ 7 protektive Verhaltensweisen anzuwenden (OR = 3,29, 95 %-KI 1,48–7,28). Weiterhin weisen Befragte mit einer eingeschränkten COVID-19-bezogenen Gesundheitskompetenz ein fast 2fach erhöhtes Risiko für eingeschränktes Schutzverhalten auf (OR = 1,96, 95 %-KI 1,0–3,9). Bei gemeinsamer Berücksichtigung aller erfasster Merkmale (M2) bleiben die Assoziationen mit dem Alter und der COVID-19-bezogenen Gesundheitskompetenz stabil.

**Tab. 3:** Binär-logistische Regression für eingeschränktes Schutzverhalten (≤ 7 Schutzverhalten)

	M1a–M1e		M2	
	OR	95 %-KI	OR	95 %-KI
*Geschlecht*				
Weiblich	1,00	–	1,00	–
Männlich	1,78	0,90–3,51	2,08	0,94–4,34
*Alter*				
≥ 50 Jahre	1,00	–	1,00	–
30 bis 49 Jahre	1,36	0,54–3,42	1,20	0,40–3,54
≤ 29 Jahre	**3,29**	**1,48–7,28**^a^	**3,47**	**1,09–11,10**^b^
*Schulbildung*				
Abitur/Fachhochschulreife	1,00	–	1,00	–
Realschule	0,58	0,26–1,30	0,86	0,34–2,14
Hauptschule	0,49	0,10–2,30	0,63	0,12–3,40
*Anzahl der gesundheitlichen Risiken*				
0 Risiken	1,00	–	1,00	–
1 Risiko	0,93	0,45–1,95	1,64	0,68–3,93
≥ 2 Risiken	0,41	0,16–1,05	0,80	0,22–2,89
*Gesundheitskompetenz*				
Ausreichend	1,00	–	1,00	–
Eingeschränkt	**1,96**	**1,0–3,90**^b^	**2,59**	**1,22–5,49**^b^

M1a: Geschlecht (*n* = 193), M1b: Alter (*n* = 203), M1c: Schulbildung (*n* = 203), M1d: Anzahl der Risiken (*n* = 203), M1e: Gesundheitskompetenz (*n* = 197); M2: M1a–M1e (*n* = 195)

*OR* Odds Ratios, *KI* Konfidenzintervall

Fettdruck: signifikantes Ergebnis: ^a^*p* < 0,01; ^b^*p* < 0,05

## Diskussion

Das erste Ziel dieser Forschungsarbeit war es, Unterschiede im protektiven Verhalten zum Schutz vor SARS-CoV‑2 von Personen mit und ohne gesundheitliche Vulnerabilität festzustellen. Von den *n* = 210 Personen Befragten gaben 72 % eine Zugehörigkeit zu höchstens einer Risikogruppe an. Die am häufigsten genannten Vulnerabilitätsfaktoren waren Alter über 50 Jahre (33 %) und eine bestehende Grunderkrankung (32 %). Über alle Teilnehmenden hinweg war ein hohes Maß der Einhaltung empfohlener Infektionsschutzmaßnahmen erkennbar. Besonders gilt dies für das Vermeiden direkter Sozialkontakte wie Feiern oder Reisen, der Verzicht auf Händeschütteln, das Einhalten von 1,5 m Mindestabstand und das Tragen eines Mund-Nasen-Schutzes (jeweils ≥ 88 %). Grundsätzlich ließ sich ein hohes Maß der Einhaltung von Maßnahmen zum Infektionsschutz auch in weiteren deutschen Studien feststellen [2, 3, 8, 18]. Entgegen der Erwartung zeichnen sich in den stratifizierten Analysen nach gesundheitlichen Risikofaktoren lediglich tendenzielle, jedoch nicht signifikante Unterschiede in der Anwendung von Schutzverhaltensmaßnahmen ab. So geben etwa 75 % der Befragten mit keinem oder maximal einem gesundheitlichen Risikofaktor an, häufig oder immer ≥ 8 Schutzverhaltensweisen anzuwenden, während der Anteil bei Personen mit ≥ 2 Vulnerabilitätsfaktoren bei etwa 87 % liegt. Zu gegensätzlichen Befunden kommen hingegen Yildirim et al. in ihrer Studie: Türk*innen, die sich als vulnerabel in Bezug auf eine Infektion mit SARS-CoV‑2 einschätzten, gaben eine höhere Bereitschaft zur Anwendung protektiver Verhaltensweisen an [21].

Ein zweites Ziel dieser Forschungsarbeit bestand darin, Zusammenhänge zwischen dem Schutzverhalten und soziodemografischen Faktoren, der gesundheitlichen Vulnerabilität sowie der COVID-19-bezogenen Gesundheitskompetenz aufzuzeigen. Hinsichtlich der gesundheitlichen Risikofaktoren, des Geschlechts und der Schulbildung konnten keine signifikanten Assoziationen mit dem Schutzverhalten nachgewiesen werden. Ebenso konnte in der Regressionsanalyse kein signifikanter Zusammenhang dieser Parameter und einer verringerten Compliance der COVID-19-bezogenen Schutzverhaltensweisen abgesichert werden. Die protektiven Verhaltensweisen variieren nach Alter und Gesundheitskompetenz. Personen ≤ 29 Jahre wenden signifikant seltener ≥ 8 Schutzverhaltensweisen an als Befragte ≥ 50 Jahre. Regressionsanalytisch ergibt sich für Teilnehmende bis 29 Jahre ein mehr als 3fach erhöhtes Risiko, ≤ 7 Infektionsschutzmaßnahmen häufig oder immer anzuwenden.

Internationale Studien stützen den Zusammenhang von Alter und Infektionsschutz zugunsten eines konsequenteren Schutzverhaltens der Älteren [10, 21]. Auch deutsche Untersuchungen kamen zu diesem Ergebnis [2, 3]. Dies kann mit der geringeren Wahrnehmung des eigenen Risikos und Ansteckungswahrscheinlichkeit der jungen Befragten sowie eines weniger vulnerabel wahrgenommenen Umfelds in Zusammenhang stehen. Aus diesen Gründen könnten jüngere Personen möglicherweise wenig Veranlassung sehen, sich einzuschränken. Einen solchen Zusammenhang stellen auch Schneider et al. (2021) her. Sie sehen die Wahrnehmung des eigenen Risikos und Erfahrungen mit SARS-CoV‑2 als entscheidend für das eigene Schutzverhalten [17]. Weiterhin ist das Anpassen der Erwerbssituation mit einhergehender Kontaktreduktion möglicherweise bei einigen jüngeren Befragten weniger gut möglich als bei älteren Teilnehmenden. Beispielsweise könnten es die auszuführenden Tätigkeiten nicht erlauben, im Homeoffice zu arbeiten, äußere Bedingungen wie die Präsenzpflicht in Berufsschulen sowie das Erreichen der Arbeitsstätten mit Hilfe von öffentlichen Verkehrsmitteln sind zu berücksichtigen. Abweichend zur vorliegenden Untersuchung konnten in vorangegangenen Erhebungen Zusammenhänge von Geschlecht und Bildungsgrad mit dem Infektionsschutzverhalten nachgewiesen werden [3, 21]. Hier sind die Stichprobengröße und -zusammensetzung zu berücksichtigen, welche die Vergleichbarkeit mit bestehenden Studien limitieren.

Wie bereits erwähnt ist die COVID-19-bezogene Gesundheitskompetenz ein wichtiger Parameter für das Schutzverhalten. Die überwiegende Mehrheit der Befragten der vorliegenden Erhebung (80 %) fühlt sich gut oder sehr gut über die Pandemie informiert, was aufgrund der Omnipräsenz von gesundheitsbezogenen Informationen in den deutschen Medien sowie im beruflichen und privaten Umfeld erwartbar ist. Dennoch berichten 57 % Verunsicherung durch die Dynamik der Informationen. Zur Auflösung dieser Diskrepanz kann die Gesundheitskompetenz herangezogen werden, welche die Beurteilung und die Anwendung von Informationen ermöglicht. Knapp die Hälfte der Befragten dieser Stichprobe geben eine ausreichende Gesundheitskompetenz an. Im bivariaten Vergleich ist der Anteil derer, die mindestens acht protektive Verhaltensweisen nutzen und eine ausreichende Gesundheitskompetenz aufweisen signifikant höher als unter denjenigen mit eingeschränkter Gesundheitskompetenz (*p* *<* *0,05*). In der multivariaten Analyse weisen Personen mit eingeschränkter Gesundheitskompetenz ein fast doppelt so hohes Risiko auf, höchstens sieben schützende Verhaltensweisen anzuwenden als diejenigen mit ausreichender Gesundheitskompetenz. In der multivariaten Gesamtbeurteilung ist für Personen mit eingeschränkter Gesundheitskompetenz sogar ein 2,7fach erhöhtes Risiko ermittelbar. Okan et al. stellten bereits fest, dass sich Personen mit eingeschränkter Gesundheitskompetenz häufiger von der Informationsdynamik überfordert zeigen als Menschen mit einer ausreichenden Gesundheitskompetenz [9]. Für Schweizer*innen, von denen etwas mehr als die Hälfte eine ausreichende COVID-19-bezogene Gesundheitskompetenz angibt, berichtet der Großteil wenig Probleme im Finden und Verstehen von Gesundheitsinformationen im Kontext der Pandemie. Mehr als die Hälfte der Befragten erklärt jedoch Schwierigkeiten im Einschätzen der Vertrauenswürdigkeit und zeigt sich verunsichert [5]. Besonders chronisch Erkrankte geben Probleme hinsichtlich der Infodemie an [5]. Ähnliche Gesundheitskompetenz-Niveaus und Verunsicherung konnte die Befragung einer repräsentativen Online-Stichprobe in Österreich ermitteln: knapp die Hälfte gibt eine limitierte Gesundheitskompetenz an, 87 % fühlen sich (sehr) gut informiert und 54 % verunsichert [7]. Dass eine eingeschränkte Gesundheitskompetenz auch mit geringer ausgeprägtem Schutzverhalten einhergehen kann, wurde auch in einer indischen Studie für Menschen mit chronischer Erkrankung ermittelt [6]. Für Studierende in Portugal konnte zudem ein Zusammenhang eines hohen Levels der Gesundheitskompetenz mit der Einstellung zur Anwendung protektiver Verhaltensweisen zur Vermeidung einer Infektion mit SARS-CoV‑2 hergestellt werden [19]. Schließlich berichten arbeitssuchende Österreicher*innen im Alter von 16 bis 29 Jahren mit niedrigem formalem Bildungsstand ausgeprägte Schwierigkeiten hinsichtlich der COVID-19-bezogenen Gesundheitskompetenz und gelten als besonders gefährdet für eine Infektion [7].

### Limitationen

Die Erhebung erfasste die subjektiven Wahrnehmungen und Selbsteinschätzungen eines Convenience Samples von Erwachsenen etwa acht Monate nach Aufkommen von SARS-CoV‑2 in Deutschland. Objektives Wissen und tatsächliches Verhalten wurden nicht überprüft. Verzerrungen z. B. zum Schutzverhalten können infolge von Recall-Bias oder sozialer Erwünschtheit nicht ausgeschlossen werden. Neben dem Querschnittscharakter der Untersuchung handelt es sich hierbei um eine selbstselektive Stichprobe, wodurch kausale Rückschlüsse und generalisierte Aussagen nicht möglich sind. Mit dieser Befragung wurden vor allem junge, gebildete, medienaffine Personen erreicht. Dies liegt insbesondere in der Verbreitung über soziale Medien begründet. Aufgrund der Erhebungsmethode ergibt sich eine selektive Stichprobe in zwei Richtungen: Einerseits wurden junge, formal gebildete, im Umgang mit wissenschaftlichen Erkenntnissen versierte Personen erreicht. Zum anderen konnten Daten von gesundheitlich vulnerablen Personen gewonnen werden.

Schließlich ist die Stichprobe mit *n* = 210 Befragten vergleichsweise gering. Zwar wurden verschiedene Strategien zur Erhöhung der Stichprobengröße angewendet (z. B. verschiedene Reminder, niedrigschwellige Kommunikation), dennoch konnten nicht mehr Teilnehmer*innen gewonnen werden. Dabei ist zu berücksichtigen, dass v. a. in der Anfangsphase der COVID-19-Pandemie zahlreiche Studien durchgeführt wurde, womit eine Ermüdung potenzieller Befragter als wahrscheinlich angesehen werden kann. Zudem konnten aufgrund begrenzter Ressourcen keine „incentives“ zur Erhöhung der Teilnahmemotivation angeboten werden. Die Stichprobengröße sowie z. T. fehlende Varianz (z. B. in der Anzahl der angewendeten Schutzverhaltensweisen) erschweren schließlich Subgruppenvergleiche. Daher ist weitere Forschung notwendig, um die Ergebnisse dieser Arbeit zu vertiefen.

## Schlussfolgerungen

Im Rahmen dieser Arbeit wird ein Vergleich von Schultzverhaltensweisen zwischen Personen mit und ohne gesundheitlichen Vulnerabilitäten sowie differenziert nach soziodemografischen und COVID-19-bezogenen Faktoren vorgenommen. Insbesondere im direkten Vergleich von Personen mit und ohne gesundheitliche Risikofaktoren findet sich bisher wenig Evidenz. Es konnten für die empfohlenen und erhobenen Maßnahmen zum Infektionsschutz hohe Zustimmungswerte ermittelt werden. Das galt für alle untersuchten Altersgruppen, Bildungsgrade und gesundheitlichen Risikofaktoren. Es konnten keine Unterschiede nach gesundheitlicher Vulnerabilität festgestellt werden. Als eine entscheidende Grundlage für die Anwendung präventiver Verhaltensweisen konnte die Gesundheitskompetenz identifiziert werden. Eine eingeschränkte Gesundheitskompetenz sowie ein niedriges Alter (unter 30 Jahre) wurden als bedeutsame Prädiktoren für ein geringer ausgeprägtes Schutzverhalten ermittelt.

## Fazit für die Praxis

- Aufgrund der begrenzten Befundlage sind zukünftig vermehrt Studien unter Berücksichtigung von Menschen mit unterschiedlicher Ausprägung gesundheitlicher Vulnerabilitäten durchzuführen. Eine Möglichkeit bestünde darin, gesundheitliche Vulnerabilitätsfaktoren in bestehende, repräsentative Erhebungen aufzunehmen.
- Aufgrund der Bedeutung der Gesundheitskompetenz für protektives Verhalten, sind Maßnahmen zur Stärkung der Gesundheitskompetenz unabhängig vom Bildungsstand umzusetzen. Dazu wäre eine frühzeitig im Lebensverlauf einsetzende Förderstrategie unter Berücksichtigung zentraler Settings von Menschen (z. B. Schule, Kommune, Arbeitsplatz) sinnvoll [16].
- Trotz der hohen Umsetzung protektiver Verhaltensweisen sollte die Bedeutung des individuellen Schutzverhaltens in verständlicher Form und an die Bedürfnisse der jeweiligen Zielgruppe angepasst kommuniziert werden (z. B. Gesundheitsinformationen in Leichter Sprache, in verschiedenen Sprachen und ggf. auditiv sowie visuell begleitet).
- Es ist zu berücksichtigen, dass individuelle Gesundheitskompetenz auch das Ergebnis struktureller Rahmenbedingungen ist, womit Settings wie Schulen oder Gesundheitsversorgungseinrichtungen zu gesundheitskompetenten Organisationen zu entwickeln sind.
